# Translational medicines “Ecosystem”

**DOI:** 10.1186/s12967-020-02325-9

**Published:** 2020-04-06

**Authors:** Vijay Mahant

**Affiliations:** MediLite Diagnostika, Inc, San Diego, USA

## Aim and scope

This section aims to provide a forum for the topics spanning the translational research ecosystem beyond academia. The section will include both research and opinions about the role of patients, government policies, healthcare providers, bio-industries such as biotechnology and pharmaceutical companies, regulatory agencies, ethics, payers, investors and academia in advancing novel medicines. Particular focus area will include opportunities and challenges in collaborating with industry and the role of stakeholders in the translational research “ecosystem”.

Translational medicine is an ecosystem, connecting a group of independent but interrelated stakeholders to promote advances in healthcare. It is comprised of patients, academic and industrial research and development professionals, commercialization teams, investment capital, regulatory agencies which enforce government policies, ethics and health insurance payers. These stakeholders often have conflicting goals and objectives and are operating within an evolving ethical framework. In today’s world that is so interconnected by technology, the new ideas and advances in healthcare echo across disciplines to create an extensive and interrelated system.

The primary role of medicine and health care organizations is to benefit patient health, including longevity, quality of life and affordability. Historically, drug discovery often has had roots in academic institutions [1]. Some of the best examples of collaborations between academia and industry in the realm of drug discovery include Copaxone, Emtriva and Taxol. Research and commercialization platforms have become the primary catalysts for funding, with investment as a driver of the ecosystem.

Funding sources for the bio-industry include private or government grants, venture capital, private investors, corporate partnerships, public capital markets (IPOs), philanthropists, charity organizations and private foundations. However, newly established bio-industry companies such as biotechnology and pharmaceutical companies are often caught in the “Valley of Death” [2] phase—the critical and challenging transition from developing a promising drug to securing funding for continued development and validation of its therapeutic and commercial potential. Navigating the “Valley of Death” is an integral part of the learning experience and can be rewarding if the process is managed successfully and effectively with a well-seasoned management team.

Many young bio-industry companies facing the “Valley of Death” phase saw an opportunity to mitigate these challenges when the “Right-to-Try” Act [3] was passed by the U.S. Congress and signed by President Trump in May 2018. Right-to-Try laws were created with the intention of allowing terminally ill patients who have failed standard-of-care treatment to try experimental therapies (drugs, biologics, devices) that have completed at least Phase I testing of the Food and Drug Administration (FDA) regulatory process. The impact and the outcome of these laws on the ecosystem is too early to predict. The risks associated with the “Right to Try” experimental drugs may be mitigated when the experimental drug is combined with an approved drug or the drug has gone through further studies such as the Phase II approval process. Funding may be further impacted by one or more factors such as the approval or disapproval of a drug by the FDA. Implementation of government policies such as the “Patient Protection and Affordable Care Act” (PPACA) will inherently impact one or more parts of the ecosystem.

According to the upper echelon theory of management [4], the beliefs and background of chief executives affect the strategic choices and outcomes of their organizational collaborations. Cultural differences between academic institutions and bio-industries can include trust, intellectual property ownership and compensation. These challenges can be mitigated by cultivating and nurturing a flourishing relationship between academia and bio-industries with effective communication, transparency, trust, and confidence. Another challenge that academia often faces is the “over visionary syndrome”. This may have less impact on bio-industries which must consider capital risk, market potential, time to commercialization, regulatory issues and reimbursement issues.

Pharmaceutical industries have recently undergone a paradigm shift and initiated “science hubs” with academic institutions to accelerate biotechnology innovation. Examples of discovery programs [5, 6] include GSK’s Tres Cantos Lab Foundation, Pfizer’s Centers for Therapeutic Innovation, Lily’s Phenotypic Drug Discovery Initiative and Merck’s SAGE Bionetworks and Clinical and Translational Science Awards Program. Academic institutions have reciprocated by establishing translational research centers such as the University of Pennsylvania’s Institute for Translational Medicine and Therapeutics (ITMAT), Stanford University’s SPARK, Harvard University’s Catalyst program and The Fred Hutchinson/University of Washington Cancer Consortium. Translational research has gained further momentum and has been expanded to harness expertise by partnering with bio-industries manufacturing consortiums such as NIIMBL (National Institute for Innovation in Manufacturing).

Strategic and cross-functional collaborations between academic institutions and bio-industries have been gaining momentum over the last decade due to the mutually beneficial and synergistic values each party brings to the table. One of the primary goals for these collaborations is improving scientific knowledge about diseases, drugs, and their pathways, as well as finding ways to apply this knowledge in a clinical setting to benefit patients’ health, longevity, quality of life and affordability. Some of the synergistic values academic institutions bring to the collaborations include credibility, wealth of knowledge and experience in early stage research, intellectual property, and lower personnel costs because many researchers are graduate students and post-graduates whose primary goal is publication or securing funding for additional academic research. On the other hand, the goals for the bio-industries include reduction of drug development costs, successful clinical trials, reduction of time to commercialization, competitive advantage, strong intellectual property position, and profitability, with the overall objective to create a “block buster” drug with a large world-wide market potential. These collaborations also have resulted in increasing high impact co-publications.

The alliance trend amongst stakeholders is global as exemplified by the Experimental Cancer Medicine Centre (ECMC) [7] based in the UK. The ECMC helps bio-industries develop cancer drugs through strategic and functional partnerships with a team of world-class scientists and clinicians focused on delivering drugs for early phase clinical trials. While most of the collaborations involve academia and bio-industries, the signing of the collaboration between Mayo Clinic (USA) and Enterprise Ireland in 2014, for economic development and job creation in Ireland [8], presented an alternative partnership structure. Global collaborations among bio-pharma companies have also been evolving to become alliances which cover the range of drug development from research initiatives to the co-marketing of drugs (such as Lipitor) and are exemplified by partnerships between Pfizer, Yamanouchi and Almirall-Prodesfarma and Menarin.

Some of the “quantum” transformations in health care and collaborations in the future are envisioned in artificial intelligence, machine learning, “big data” [9], data refineries, quantum computing, encryption of patient’s personal data using “quantum entanglement”, and the emergence and growth of specialized branches of medicine such as space medicine. The orchestration and integration of multi-disciplinary sciences, engineering, financing, regulations, and medicine will play a critical role in anchoring and shaping the shared vision and goal of improving healthcare, longevity and quality of life at an affordable cost. The participation of experts in the ecosystem is welcome and encouraged to contribute to advancing translational medicine for a common mission, vision and goal.
